# Effectiveness of Bee Venom Injection for Parkinson’s Disease: A Systematic Review

**DOI:** 10.3390/toxins17040204

**Published:** 2025-04-20

**Authors:** Hyein Jeong, Kyeong Han Kim, Seong-gyu Ko

**Affiliations:** 1 Department of Science in Korean Medicine, College of Korean Medicine, Kyung Hee University, Seoul 02446, Republic of Korea; frogcream@gmail.com; 2 Department of Preventive Medicine, College of Korean Medicine, Woosuk University, Jeonju 55338, Republic of Korea

**Keywords:** Parkinson’s disease, bee venom, bee venom acupuncture, systematic review

## Abstract

Parkinson’s disease (PD) is the second most common neurodegenerative disorder worldwide, affecting over 8.5 million people as of 2019. While standard pharmacological treatments help alleviate symptoms, their long-term use can lead to side effects such as dyskinesia. Bee venom acupuncture (BVA) involves the use of a natural toxin derived from bees that can be used for pain relief and treating neurological disorders. This study aimed to review the efficacy and safety of BVA for the treatment of PD. This review protocol was prospectively registered with PROSPERO (CRD420251000577). We searched eight databases in February 2025 and selected 12 studies involving 215 PD patients treated with BVA. Idiopathic Parkinson’s disease (IPD) is the most common diagnosis. The concentration and dosage per session ranged from 0.03 to 0.1 mg/mL and from 0.1 to 1.0 mL, respectively. Twenty-four different outcome measures were used, with the Unified PD Rating Scale employed in 91.7% of the studies. All studies reported improvements in outcomes. Mild adverse effects such as swelling and itching were noted in four studies (33.3%); however, no severe reactions such as anaphylactic shock occurred. These findings suggest that BVA has the potential for broader clinical applications in the treatment of PD.

## 1. Introduction

Parkinson’s disease (PD) is the second most common neurodegenerative disorder globally, affecting over 8.5 million individuals as of 2019 [1]. In the United States, approximately one million people live with PD, and ~90,000 new cases are diagnosed annually [2].

Standard PD treatment strategy involves medications, such as levodopa, dopamine agonists, and MAO-B (Monoamine Oxidase B) inhibitors [1]. However, long-term use of these pharmacotherapies can lead to motor complications and dyskinesia, and some patients may experience insufficient symptom control or adverse effects that necessitate discontinuation.

Recent studies have explored alternative therapies, including acupuncture and bee venom acupuncture (BVA), as adjunctive treatments for PD. BVA is a widely used animal-derived toxin that is particularly common in East Asian countries, such as Korea, where it is processed into injectable forms [3]. Although it has demonstrated strong analgesic and anti-inflammatory effects and is widely applied under various conditions [4,5], there is a risk of anaphylaxis, a potentially fatal allergic reaction [6].

A pilot study demonstrated that both acupuncture and BVA showed promising results in improving motor function and quality of life in patients with PD [7]. Another study suggested that BVA may exert neuroprotective effects owing to its anti-inflammatory properties [8]. A previous systematic review examined the effects of acupuncture on idiopathic Parkinson’s disease (IPD) [9]. However, only three studies were included, one of which included BVA; it was 8 years old and needed to be updated. Therefore, this review aimed to assess whether bee venom injection is an effective and safe treatment for managing Parkinson’s disease.

## 2. Results

### 2.1. Study Description

**Figure 1:**
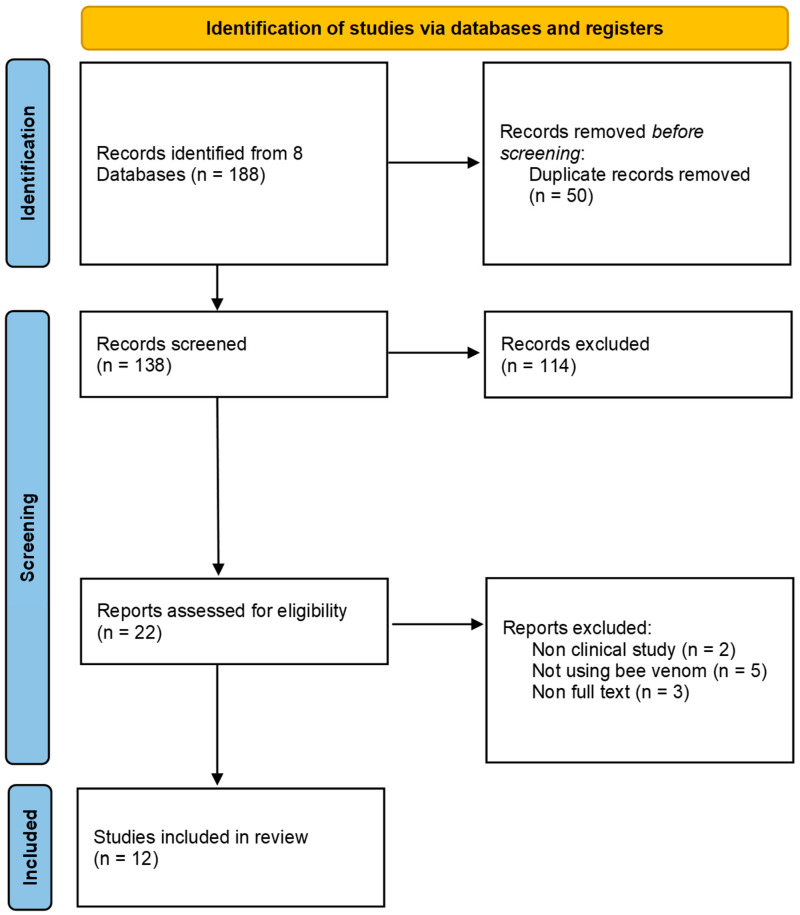
PRISMA flow chart.

We selected 12 studies [7,8,9,10,11,12,13,14,15,16,17,18] that met our inclusion criteria (Figure 1 and Table 1).

**Figure 2:**
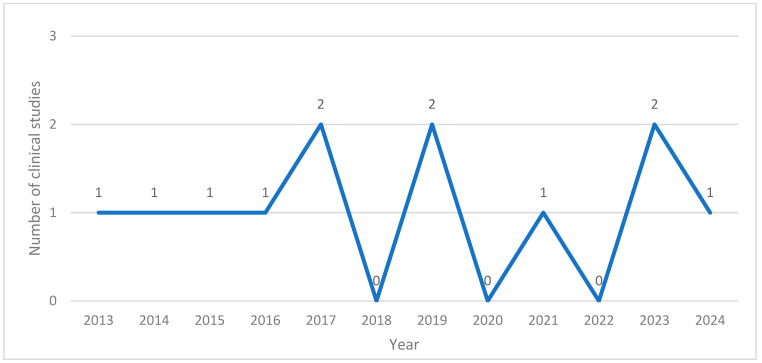
Number of clinical studies published per year.

The application of BVA in research in South Korea was first documented in 2013. Between 2013 and 2024, the annual number of related publications ranged from none to two. Notably, no studies were published in 2018, 2020, or 2022. There were six case reports [12,13,15,16,17,18], one retrospective analysis [11], one prospective open-label investigation [10], and four randomized controlled trials (RCTs) [7,8,14,19] (Figure 2).

### 2.2. Medical Conditions

Twelve distinct clinical conditions were identified in 12 reviewed studies. They were grouped into three categories: IPD, PD with specific clinical characteristics, and secondary forms of Parkinsonism. IPD was the most explored condition, occurring in nine studies (75.0%) [8,9,10,12,13,14,15,18,19]. Two studies (16.7%) specifically focused on PD with additional features, such as postural instability, gait impairment, and camptocormia [11,16]. Secondary Parkinsonism, which includes conditions such as drug-induced Parkinsonism, was the subject of only one study (8.3%) [17].

### 2.3. Sample Size

A total of 215 participants were involved in the 12 articles. Sample size varied significantly between the studies, with the smallest involving a single participant and the largest including 63 patients.

### 2.4. Overview of BVA Treatment

BVA therapy was delivered via syringe-based injections into specific acupuncture points, with the concentration of the solution tailored according to the type of Parkinsonian condition. For individuals diagnosed with IPD, BV concentration ranges from 0.05 to 0.1 mg/mL. In these cases, the volume administered per session varied from 0.1 to 1.0 mL, with the cumulative dosage across sessions falling between 1.1 and 24 mL. In patients presenting with PD accompanied by distinct symptoms, such as postural instability or camptocormia, a standardized concentration of 0.05 mg/mL was used. The per-session volumes ranged from 0.2 to 1.0 mL, and the total administered dose was between 8.4 and 24 mL. For those with drug-induced Parkinsonism, treatment was conducted at lower concentrations, between 0.03 and 0.05 mg/mL. Each session involved a 1.0 mL injection, with the entire course amounting to 19 mL. However, there have been several instances where specific dosage data were not disclosed. In one study that used BVA named ‘eBV’ for PD [13], the venom concentration was not reported. In another case, ref. [10] the per-session dosage was not specified. Furthermore, the total dosage administered was omitted in two studies, one involving general Parkinson’s, and the other in which the number of sessions was not documented [12,13] (Table 2).

### 2.5. Outcome Measures

#### Clinical Outcomes

**Figure 3:**
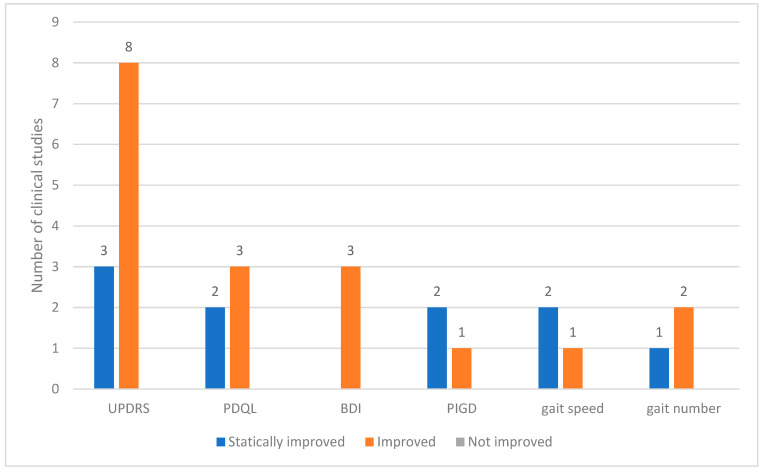
Outcomes used in clinical studies on bee venom acupuncture for Parkinson’s Disease. Abbreviations: BDI, Beck Depression Inventory; PDQL, Parkinson’s Disease Quality of Life Questionnaire; PIGD, postural instability and gait disorder; UPDRS, Unified Parkinson’s Disease Rating Scale.

Across the 12 clinical studies reviewed, 24 distinct outcome measures were analyzed. These outcomes were categorized based on the degree of change observed as statistically improved, improved, or not improved. The Unified PD Rating Scale (UPDRS) is a commonly used assessment tool. Among the studies utilizing the UPDRS, eight reported general improvements and three demonstrated statistically significant improvements. Other frequently assessed indicators included the PD quality of life questionnaire (PDQL) and Beck depression inventory (BDI). In all instances in which these tools were applied, the outcomes showed consistent improvements. Measures related to motor function, such as postural instability and gait disorder (PIGD), gait speed, and gait step count, also showed improved results (Figure 3).

### 2.6. Summary of Adverse Events

**Figure 4:**
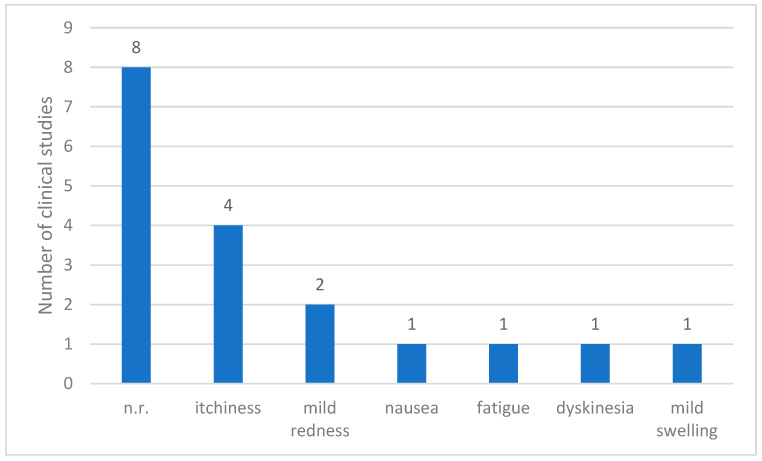
Reported adverse events in clinical studies on Parkinson’s Disease. Abbreviations: n.r.: not reported.

Of the 12 clinical studies reviewed, adverse events were not reported in 8 studies. However, the remaining four studies [7,8,9,10] provided records of the side effects associated with treatment. The most commonly observed adverse reaction was itching, which occurred in four patients. Mild skin redness was noted in two patients, whereas nausea, fatigue, dyskinesia, and mild swelling were reported once. Overall, the reported adverse effects were generally minor and localized. Most adverse events involved temporary irritation or discomfort at the injection site, with no evidence of serious systemic complications (Figure 4).

### 2.7. Types of Co-Interventions Used in Clinical Studies on PD

**Figure 5:**
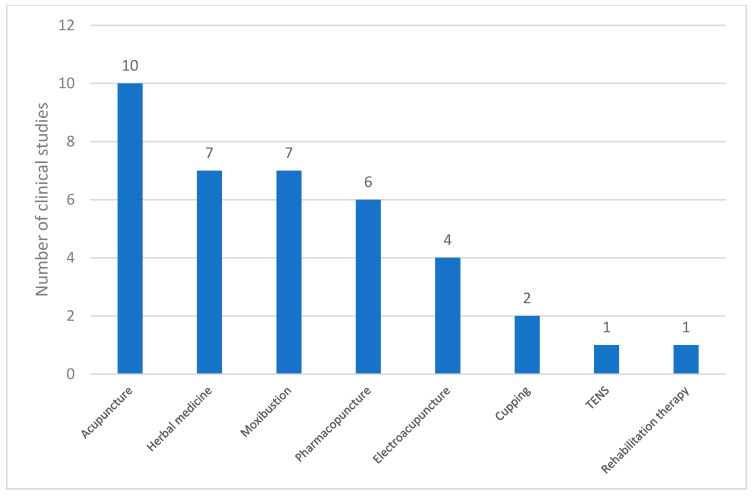
Types of co-interventions used in clinical studies on PD. Abbreviations: TENS, transcutaneous electrical nerve stimulation.

In the 12 studies that incorporated co-interventions, various complementary therapies were used. The data presented in Figure 5 illustrate the frequency of these co-interventions. Acupuncture was the most commonly employed co-intervention (n = 10), followed by herbal medicine (n = 7) and moxibustion (n = 7). Pharmacopuncture (n = 6) and electroacupuncture (n = 4) were frequently used. Less frequently reported interventions included cupping therapy (n = 2), TENS (n = 1), and rehabilitation therapy (n = 1). These findings indicate that acupuncture-based therapies were the predominant co-interventions in the analyzed studies, and other techniques, such as herbal medicine and moxibustion, also played a significant role.

## 3. Discussion

BVA is an alternative therapy that combines traditional acupuncture techniques with the injection of purified, diluted bee venom into specific acupuncture points. The main component of BVA is melittin, along with allergenic substances such as protease inhibitors and peptides [20]. While these components may have therapeutic effects, they can also cause side effects and allergic reactions [21]. Although anaphylactic reactions are rare, they can still occur and may lead to fatal outcomes [6].

PD is a debilitating neurodegenerative disease. Despite the availability of pharmacological treatments, they are not effective in all patients and may cause significant adverse effects. Consequently, alternative treatment options such as BVA have been explored for PD management. This study aimed to evaluate the applicability and safety of BVA for the treatment of PD.

The application of BVA in PD has been steadily published since 2013, in contrast to studies applying BVA to other conditions that began in the early 2000s [4,5,22]. A review of BVA in PD animal models noted a marked increase in studies since 2010, with more than 20 papers published annually and more than 50 published by 2016 [23]. Although BVA has traditionally been used for its anti-inflammatory and analgesic effects in joint and pain-related conditions, its application is now expanding to neurological diseases such as PD.

Among the 12 studies reviewed, 4 were RCTs, while the remaining included case reports (6), one retrospective analysis, and one prospective open-label investigation. Notably, RCTs will continue until 2023, indicating an ongoing effort to establish higher levels of evidence for the use of BVA in PD.

In the PD, the BVA concentration generally ranged from 0.03 to 0.1 mg/mL. While some studies did not specify the exact concentration used per session, the manufacturers (e.g., eBV made by Jaseng) did not disclose the precise dilution ratio. Compared to concentrations used for neck pain (0.05–0.5 mg/mL) and shoulder pain (0.005–1.0 mg/mL), BVA for PD is typically used at concentrations from 5 to 10 times lower. This is likely because most BVA indications involve musculoskeletal conditions, in which high-concentration injections directly stimulate the affected muscles or ligaments. In contrast, PD is a neurological condition. Therefore, the therapeutic focus is on stimulating specific acupuncture points using a standard clinical concentration, typically approximately 0.05 mg/mL.

The volume per session ranged from 0.2 to 1.0 mL, which is comparable to dosages used in neck pain (0.1–1.0 mL) and shoulder pain (0.1–2.0 mL). In Korean clinical practice, practitioners often use insulin syringes to draw insulin from BVA vials, with a maximum volume of 1.0 mL per treatment. As injections are distributed across multiple acupuncture points, the volume per point is typically approximately 0.1 mL.

Twenty-four outcome measures were used in the twelve included studies. Except for one study, the remaining 11 used the UPDRS, and all reported improvements. Other commonly used tools include the PDQL, BDI, and PIGD scores, and gait parameters such as speed and step count.

The UPDRS [24] is a comprehensive tool for assessing both motor and non-motor symptoms of PD and comprises four parts. Part 3 of UPDRS evaluated motor functions, including tremor, rigidity, and balance. Most studies focused on parts 2 and 3, whereas only five studies [7,8,12,13,14] used the full version, including parts 1 and 4. Notably, all were RCTs except for two studies [12,13] that were RCTs. Given the length and complexity of the full UPDRS, it is likely that partial versions were used in case-based studies to reduce evaluation time.

The PDQL [25] was employed to assess the quality of life in patients with PD, and a similar tool, the PDQ-39, was used in one study [8]. These instruments are important because patients with PD generally report a poorer physical and mental quality of life than healthy controls [26]. Since PD remains incurable, improving the overall quality of life may be a more realistic treatment goal than symptom relief alone. Studies on consensus outcome measures for PD recommend the MDS-UPDRS as the standard tool for both motor and non-motor assessments and suggest using the shortened PDQ-8 version for quality-of-life evaluation [27]. Notably, most studies included in this review used approximately 40-item tools, which were longer than the PDQ-8. The original version was used instead of the shortened PDQ-8, as a longer version of the questionnaire can provide more detailed and comprehensive information, if feasible.

BVA-related adverse effects were reported in four studies. These symptoms were all mild, including itching, burning sensations, and localized pain, with no cases of severe reactions such as anaphylaxis. This may be attributed to the routine use of skin tests before treatment in most studies and the exclusion of participants with positive skin test results from the RCTs.

Except for one study [17], all other studies included the concomitant use of Western medications and other complementary therapies, such as acupuncture, herbal medicine, and moxibustion. While most studies have reported favorable outcomes without serious side effects, the lack of studies specifically focusing on safety remains a limitation.

This review has some limitations. It is unclear whether the absence of adverse event reports in some studies is due to the actual absence of such events or underreporting. Therefore, accurately assessing the safety profile of BVA remains challenging. However, according to a previous meta-analysis on BVA, the estimated incidence of severe reactions, such as anaphylaxis, is relatively low (0.045%) [6]. Of the twelve included studies, six were case reports and four were RCTs. More RCTs are required to improve the level of evidence. Most studies have combined BVA with other treatments and Western medicine, making it difficult to isolate the specific effects of BVA. Several case reports have been published in Korea, where BVA is commonly used alongside acupuncture or moxibustion in clinical practice. Therefore, future high-quality RCTs should assess the efficacy of BVA as a standalone therapy.

## 4. Conclusions

BVA shows promising potential as an adjunct treatment alongside conventional Western medicine for the disease. Large-scale multicenter trials and randomized controlled studies are warranted to further validate its efficacy and safety.

## 5. Materials and Methods

### 5.1. Search Methods for Identification of Studies

This systematic review was registered in the PROSPERO International Prospective Register of Systematic Reviews (registration number: CRD420251000577). The following databases were searched from their inception until February 2025: PubMed, Embase, Cochrane Library, Chinese National Knowledge Infrastructure (CNKI), ScienceON (https://scienceon.kisti.re.kr), KISS (https://kiss.kstudy.com/), RISS (http://www.riss.or.kr), and OASIS (https://oasis.kiom.re.kr). Studies involving human subjects, including RCTs, case reports, and cohort studies on bee venom injection for Parkinson’s disease, will be included without language restrictions. The search strategy for each database is presented in Supplementary File S1.

### 5.2. Study Selection

#### 5.2.1. Types of Studies

Studies involving human subjects, including RCTs, case reports, and cohort studies on bee venom injections for PD, were included.

#### 5.2.2. Types of Participants

We included trials in which participants were diagnosed with PD. The study population included individuals of all ages, races, and sexes.

### 5.3. Types of Intervention

#### 5.3.1. Experimental Interventions

The experimental group received BVA acupuncture. BVA involves the injection of diluted bevacizumab into specific acupuncture points associated with PD management.

#### 5.3.2. Control Interventions

The control group received standard Western medical treatment for PD, which may include medications such as levodopa or dopamine agonists. Alternatively, the control group may have received traditional body acupuncture therapy without bee venom. Studies comparing combined BVA and Western medicine versus Western medicine alone were excluded to isolate the effects of BVA.

### 5.4. Data Collection and Analysis

#### Data Extraction and Management

Two independent reviewers conducted this study. They assessed the studies for inclusion by reviewing titles, abstracts, and full texts to determine their eligibility based on predefined criteria. Any disagreements or discrepancies between the two reviewers were resolved through discussion or by involving a third reviewer. The entire screening process was documented and presented using the Preferred Reporting Items for Systematic Reviews and Meta-Analyses (PRISMA) flow diagram, which provides a transparent overview of the study selection process.

## Figures and Tables

**Table 1:** Characteristics of included clinical studies.

Research	Year	Study Design	No of Participants	Disease	Acupoint	Concentration (Dosage)	Outcome Measure	Main Result	Adverse Evenets	Co-Intervention	Western Medicine
Doo2015 [10]	2015	Prospective Open-Label Study	11	Idiopathic Parkinson’s disease	(bilateral) GB20, LI4, GB34, ST36, LR3	1. Concentration: 0.05 mg/mL 2. 1 session: n.r mL 3. Total 24 session: n.r mL	1. UPDRS II, III 2. PDQL 3. BDI 4. Postural stability (MXE, DCL) 5. walk 20-m 5-1. 20-m gait speed 5-2. 20-m gait number 6. Symptom change	1. positive ^a^ 2. positive ^a^ 3. improved 4. improved 5-1. positive ^a^ 5-2. improved 6. improved	mild pain, slight bleeding (acupuncture) mild redness, itchiness(bva)	acupuncture	o
Hartmann2016 [8]	2016	RCT	40 (I 20/C 20)	Parkinson’s Disease	subcutaneous	1. Concentration: 0.1 mg/mL 2. 1 session: 0.1 mL 3. Total 11 session: 1.1 mL	1. UPDRS 1-4 2. H-Y stage 3. Schwab and England scores 4. PDQ-39 5. MRI 6. [123I]-FP-CIT SPECT 7. BREF 8. MMSE 9. LED	1. improved 2. improved 3. improved 4. improved 5. improved 6. improved 7. improved 8. improved 9. improved	light adverse effects: redness/itching at the injection site (placebo: 6/bee venom: 165), insomnia (placebo: 1/bee venom: 1), nausea (placebo: 9/bee venom: 3), fatigue (placebo: 10/bee venom: 2), dyskinesia (placebo: 1/bee venom: 1), bradycardia (placebo: 2/bee venom: 0).	x	o
Cho2013 [7]	2013	RCT	35(Atx 13, BVA 13, C 9)	Idiopathic Parkinson’s Disease	(bilateral) GB20, LI11, GB34, ST36, LR3	1. Concentration: 0.05 mg/mL 2. 1 session: 1.0 mL 3. Total 16 session: 16 mL	1. UPDRS 2. PDQL 3. BDI 4. BBS 5. Walk 30-m 5-1. 30-m walking time 5-2. Steps to walk 30-m	1. positive ^a^ 2. improved 3. improved 4. positive ^a^ 5-1. positive ^a^ 5-2. improved	Itchiness (bva, 1)	x	
Yang2017 [11]	2017	Retrospective Study	23 (IPD 16, Atypical Parkinsonism 7)	Postural Instability and Gait Difficulty in patient	(bilateral) GB20, LI11, GB34, ST36, LR3	1. Concentration: 0.05 mg/mL 2. 1 session: 1 mL 3. Total 19 session: 19 mL	1. PIGD score (based on UPDRS)	1. positive ^a^	n.r	1. Acupuncture 2. Herbal medicine 3. Elecroacupuncture 4. Pharmacopuncture 5. Moxibustion 6. Cupping	o
Cho2017 [9]	2017	RCT	63 (BVA 24, sham 24, C 15)	Idiopathic Parkinson’s Disease	(bilateral) GB20, LI11, GB34, ST36, LR3	1. Concentration: 0.05 mg/mL 2. 1 session: 1 mL 3. Total 24 session: 24 mL	1. UPDRS 2,3 2. PIGD 3. PDQL 4. BDI 5. Walk 20-m 5-1. 20-m gait speed 5-2. 20-m gait number 6. Postural Stability 6-1. MXE 6-2. DCL	1. positive ^a^ 2. positive ^a^ 3. positive ^a^ 4. improved 5-1. improved 5-2. positive ^a^ 6. improved	mild pain, slight bleeding (acupuncture) mild swelling, itchiness(bva)	1. Acupuncture	o
Lee2023(1) [12]	2023	Case report	2	Parkinson’s Disease	(bilateral) GB20, GB20 under 2 chon, LI11, GB34, ST36	1. Concentration: 0.05 mg/mL 2. 1 session: 1 mL 3. Total session: n.r	1. G-walk 2. UPDRS 3. Symptom change	1. improved 2. improved 3. improved	n.r	1. Acupuncture 2. Herbal medicine 3. Moxibusion 4. Pharmacopuncture (Jungsongouhyul)	o
Hwang2019 [13]	2019	Case report	1	Parkinson’s Disease	Huatuojiaji (Hyeopcheok)	1. Concentration: n.r (eBV by Jaseng) 2. 1 session: 0.2-0.4mL 3. Total session: n.r	1. H-Y stage 2. UPDRS 3. BBS 4. GSRS 5. NRS (Symptom) 6. Stress index(uBioMacpa) 7. PDQL	1. improved 2. improved 3. improved 4. improved 5. improved 6. improved 7. improved	n.r	1. Acupuncture 2. Herbal medicine 3. Moxibusion 4. Pharmacopuncture (Homince Placenta)	o
Lee2023(2) [14]	2023	RCT	34(BVA 11, Sham 9, C 14)	Idiopathic Parkinson’s Disease	(bilateral) GB20, LI11, GB34, ST36, LR3	1. Concentration: 0.05 mg/mL 2. 1 session: 1 mL 3. Total 24 session: 24 mL	1. PET 2. ASL 3. UPDRS	1. improved 2. improved 3. improved	n.r	1. Acupuncture	
Kwak2024 [15]	2024	Case report	1	Idiopathic Parkinson’s disease	(bilateral) GB20, LI11, LR3, GB34, ST36	1. Concentration: 0.05 mg/mL 2. 1 session: 1 mL 3. Total 4 session: 4 mL	1. NRS 2. KPPS 3. UPDRS II, III 4. G-walk 5. Symptom change	1. improved 2. improved 3. improved 4. improved 5. improved	n.r	1. Acupuncture 2. Herbal medicine 3. Moxibusion 4. Electroacupuncture 5. TENS 6. Pharmacopuncture (Jungsongouhyul)	o
Kim2019 [16]	2019	Case report	1	Parkinson’s Disease with Postural Instability and Camptocormia	(bilateral) GB20, LI11, ST36, GB34	1. Concentration: n.r 2. 1 session: 0.4 mL 3. Total 21 session: 8.4 mL	1. UPDRS II, III 2. Duration of independent ambulation during the day 3. Symptom change	1. improved 2. improved 3. improved	n.r	1. Acupuncture 2. Herbal medicine 3. Moxibusion 4. Pharmacopuncture (Jungsongouhyul)	o
Choi2021 [17]	2021	Case report	1	Drug-Induced Parkinsonism	(bilateral) GB20, LI11, ST36, LR8, SP7	1. Concentration: 0.03–0.05 mg/mL 2. 1 session: 1.0 mL 3. Total 19 session: 19 mL	1. UPDRS II, III 2. PIGD score 3. 20m walking video 4. Symptom change	1. improved 2. improved 3. improved 4. improved	n.r	1. Acupuncture 2. Herbal medicine 3. Moxibusion 4. Electroacupuncture 5. Pharmacopuncture (Jungsongouhyul)	x
Lee2014 [18]	2014	Case report	3	Idiopathic Parkinson’s disease	(bilateral) LR3, GB34, ST36, LI11, GB20	1. Concentration: 0.05 mg/mL 2. 1 session: 1.0 mL 3. Total 18 session: 18 mL	1. UPDRS II, III 2. Symptom change	1. improved 2. improved	n.r	1. Acupuncture 2. Herbal medicine 3. Moxibusion 4. Electroacupuncture 5. Cupping 6. Rehabilitation therapy	o

^a^ *p* < 0.05.

**Table 2:** BVA concentrations and dosages according to the medical conditions of patients.

Medical Conditions of Participants	Concentration (mg/mL)	Dosage	
		Dosage Per 1 Session (mL)	Dosage for Entire Treatment (mL)
Idiopathic Parkinson’s Disease	0.05–0.1 mg/mL	0.1–1.0 mL	1.1–24.0 mL
Parkinson’s Disease with Specific Features (postural instability, camptocormia)	0.05 mg/mL	0.2–1.0 mL	8.4–24.0 mL
Drug-Induced Parkinsonism	0.03–0.05 mg/mL	1.0 mL	19.0 mL

## Data Availability

No new data were created or analyzed in this study.

## Supplementary Materials

The following supporting information can be downloaded at: https://www.mdpi.com/article/10.3390/toxins17040204/s1.

## Abbreviations

The following abbreviations are used in this manuscript:

Atx	Acupuncture
BBS	Berg balance scale
BDI	Beck depression inventory
BREF	Batterie rapide d’évaluation frontale
BVA	Bee venom acupuncture
C	Control group
DCL	Directional control
IPD	Idiopathic Parkinson’s disease
I	Intervention group
KPPS	King’s Parkinson’s disease pain scale
LED	Levodopa equivalent dose
MMSE	Mini-mental state examination
MRI	Magnetic resonance imaging
MXE	Maximum excursion
n.r	Not reported
NRS	Numerical rating scale
PD	Parkinson’s disease
PDQ-39	39-item Parkinson’s disease questionnaire
PDQL	Parkinson’s disease quality of life questionnaire
PET	Positron emission tomography
PIGD	Postural instability and gait disorder
RCT	Randomized controlled trial
TENS	Transcutaneous electrical nerve stimulation
UPDRS	Unified Parkinson’s disease rating scale
123I]-FP-CIT SPECT	Single photon emission computed tomography using [123I]-FP-CIT
