# Supplementary material for: Effectiveness of Bee Venom Injection for Parkinson’s Disease: A Systematic Review
Source: Toxins (Basel). 2025 Apr 20;17(4):204. doi: 10.3390/toxins17040204 (PMC12031087; doi:10.3390/toxins17040204)
Supplement: Supplementary file 1 [file toxins-17-00204-s001.zip › toxins-3579167-supplementary.pdf]

Table S1. Search strategy in Pubmed

NO.	Search strategy	Item
#1	("parkinson Disease"[Mesh] OR “parkinson disease”[tw] OR “parkinson’s disease”[tw])	147,744
#2	("bee venom”[tw] OR "melittin"[tw] OR “bee”[tw]) AND (“pharmacopuncture”[tw] OR “acupuncture”[Mesh] OR “acupuncture”[tw] OR “injection”[tw])	723
#3	#1 AND #2	29

Table S2. Search strategy in EMBASE

NO.	Search strategy	Item
#1	("parkinson disease"/exp OR "parkinson disease":ti,ab)	216,639
#2	('bee venom'/exp OR 'bee venom':ti,ab OR 'melittin':ti,ab OR 'bee':ti,ab) AND ('pharmacopuncture':ti,ab OR 'acupuncture'/exp OR 'acupuncture':ti,ab OR 'injection':ti,ab)	1,118
#3	#1 AND #2	39

S3. Search strategy in Cochrane

NO.	Search strategy	Item
#1	("parkinson disease":ti,ab,kw)	13,074
#2	("bee venom":ti,ab,kw OR "melittin":ti,ab,kw OR "bee":ti,ab,kw)	484
#3	("pharmacopuncture":ti,ab,kw OR "acupuncture":ti,ab,kw OR "injection":ti,ab,kw)	115,227
#4	#1 AND #2 AND #3	11

Search strategy in RISS

NO.	Search strategy	Item
#1	파킨슨|파킨슨병|파킨슨 증후군|Parkinson disease	3,175
#2	봉침|벌침|bee venom|멜리틴|melittin|약침|pharmacopuncture	3,672
#3	#1 AND #2	44

Search strategy in KISS

NO.	Search strategy	Item
#1	(봉침|bee venom) AND (Parkinson)	11

Search strategy in ScienceOn

NO.	Search strategy	Item
#1	(파킨슨|파킨슨병|파킨슨 증후군|Parkinson disease)[TI]	25,249
#2	(봉침|벌침|bee venom|멜리틴|melittin|약침|pharmacopuncture)[TI]	3,063

#3	#1 AND #2	44

Search strategy in OASIS

NO.	Search strategy	Item
#1	(파킨슨|파킨슨병|파킨슨 증후군|Parkinson disease)	134
#2	(봉침|벌침|bee venom|멜리틴|melittin|약침|pharmacopuncture)	1540
#3	#1 AND #2	1

Search strategy in CNKI

NO.	Search strategy	Item
#1	(SU='帕金森病' OR SU='帕金森氏病') AND (SU='穴位注射' OR SU='药针' OR SU='蜂针' OR SU='蜂毒' OR SU='Point injection')	16
